# Additional Haplogroups of *Toxoplasma gondii* out of Africa: Population Structure and Mouse-Virulence of Strains from Gabon

**DOI:** 10.1371/journal.pntd.0000876

**Published:** 2010-11-02

**Authors:** Aurélien Mercier, Sébastien Devillard, Barthélémy Ngoubangoye, Henri Bonnabau, Anne-Laure Bañuls, Patrick Durand, Bettina Salle, Daniel Ajzenberg, Marie-Laure Dardé

**Affiliations:** 1 IFR 145 GEIST, EA 3174 NeuroEpidémiologie Tropicale et Comparée, Université de Limoges, Limoges, France; 2 CNRS, UMR5558, Laboratoire de Biométrie et Biologie Evolutive, Université Lyon 1, Villeurbanne, France; 3 Centre de Primatologie, Centre International de Recherche Médicale de Franceville (CIRMF) BP 769, Franceville, Gabon; 4 CHU Limoges, Service de l'Information Médicale et de l'Evaluation, Unité Fonctionnelle de Recherche Clinique et de Biostatistiques, Limoges, France; 5 Centre IRD, Génétique et Evolution des Maladies Infectieuses (UMR CNRS/IRD 2724), Montpellier, France; 6 Centre National de Référence (CNR) Toxoplasmose/T. gondii Biological Resource Center (BRC), Centre Hospitalier-Universitaire Dupuytren, Limoges, France; Leiden University Medical Center, Netherlands

## Abstract

**Background:**

*Toxoplasma gondii* is found worldwide, but distribution of its genotypes as well as clinical expression of human toxoplasmosis varies across the continents. Several studies in Europe, North America and South America argued for a role of genotypes in the clinical expression of human toxoplasmosis. Genetic data concerning *T. gondii* isolates from Africa are scarce and not sufficient to investigate the population structure, a fundamental analysis for a better understanding of distribution, circulation, and transmission.

**Methodology/Principal Findings:**

Seropositive animals originating from urban and rural areas in Gabon were analyzed for *T. gondii* isolation and genotyping. Sixty-eight isolates, including one mixed infection (69 strains), were obtained by bioassay in mice. Genotyping was performed using length polymorphism of 13 microsatellite markers located on 10 different chromosomes. Results were analyzed in terms of population structure by Bayesian statistical modeling, Neighbor-joining trees reconstruction based on genetic distances, *F*_ST_ and linkage disequilibrium. A moderate genetic diversity was detected. Three haplogroups and one single genotype clustered 27 genotypes. The majority of strains belonged to one haplogroup corresponding to the worldwide Type III. The remaining strains were distributed into two haplogroups (*Africa 1* and *3*) and one single genotype. Mouse virulence at isolation was significantly different between haplogroups. *Africa 1* haplogroup was the most virulent.

**Conclusion:**

*Africa 1* and *3* haplogroups were proposed as being new major haplogroups of *T. gondii* circulating in Africa. A possible link with strains circulating in South and Central America is discussed. Analysis of population structure demonstrated a local spread within a rural area and strain circulation between the main cities of the country. This circulation, favored by human activity could lead to genetic exchanges. For the first time, key epidemiological questions were addressed for the West African *T. gondii* population, using the high discriminatory power of microsatellite markers, thus creating a basis for further epidemiological and clinical investigations.

## Author Summary

Prevalence of human toxoplasmosis in tropical African countries usually exceeds 50%. Its role as a major opportunistic infection of AIDS patients is regularly described. Due to the lack of investigation, congenital infection is certainly underestimated in Africa. Incidence of *Toxoplasma* ocular disease is higher in Africa and South America than in Europe. Severe cases in immunocompetent patients were described after infection acquired in Amazonia, but nothing is known about such cases in Africa. Several studies argued for a role of genotypes in the clinical expression of human toxoplasmosis, and for a geographical structuration of Toxoplasma across continents. Genetic data concerning isolates from Africa are scarce. Here, apart from the worldwide Type III, we described two main haplogroups, Africa 1 and 3. We detected genetic exchanges between urban centers favored by trade exchange and transportation. It shows how important human influence is, even in shaping the genetic structure of a zoonotic disease agent. Finding of identical haplogroups in South America suggested that these African and American strains share a common ancestor. As a higher pathogenicity in human of South American genotypes has been described, this similarity of genotypes should encourage further clinical studies with genotype analysis in Africa.

## Introduction

*Toxoplasma gondii* is a worldwide haploid protozoan parasite, and distribution of its genotypes varies across the continents (e.g. [1]). In Europe and the USA, *T. gondii* has a low genetic diversity with three main lineages, Type I, II and III, based on clonal population structure and virulence in mice [2], [3]. In tropical regions of South America, *T. gondii* strains are highly divergent from those of Europe or North America and display a high degree of genetic diversity [4],[5],[6],[7]. Although Type II isolates have been found in Chile and Brazil [8], [9], they seems very rare elsewhere in South America [2], [10]. Genetically distinct isolates are found in different regions of South America [11]. Common clonal lineages, different from the three classical Types, may circulate on this continent [5] with some atypical genotypes highly pathogenic to humans [6]. For example, a high frequency of ocular toxoplasmosis in some areas of Brazil [12], as well severe cases of acquired toxoplasmosis in otherwise healthy adults have been reported [4], [13].

In contrast to Europe and the Americas, the genetic diversity and population structure of *T. gondii* from Africa, where limited data are available, are still controversial. Two recent genotyping studies based on *T. gondii* strains isolated from chickens from diverse African countries [14], [15] have suggested that like in Europe and in the USA, the same three main lineages predominate in Africa with one strain considered to be a recombinant between Type II and III strains [16]. Nonetheless, non classical genotypes of the parasite, called *Africa 1* and *Africa 2*, have been isolated from immunocompromised patients with toxoplasmosis acquired in Western and Central Africa [17]. Because these genotypes were also repeatedly recovered in patients from different African countries they were proposed as common clonal lineages in Africa. It is clear that issue of the population structure of *T. gondii* in Africa is far from being resolved.

As in many African countries, Gabon has a contrasted environment with remote rural areas and urban centers which permitted analysis of genotype circulation in different biotopes. Microsatellites, as rapidly evolving neutral markers, are excellent tools for differentiating among strains and analysing population structure. In the present paper, we genotyped 69 *T. gondii* strains from domestic animals in Gabon using for the first time 13 microsatellite (MS) markers [17],[18],[19],[20] to precisely identify the strains, study the Gabonese population genetic structure and make comparison with reference strains and isolates from different continents. Haplogroups associated with Africa are described and correlated to mouse-virulence. Finally, we discuss the possible relationships between human pathogenicity, *T. gondii* genetic diversity, and population structure on the African continent.

## Methods

### Ethics statement

All procedures carried out on animals were in agreement with ethical rules. All experimental procedures were conducted according to European guidelines for animal care (“Journal Officiel des Communautés Européennes”, L358, December 18, 1986) after reviewed by the Ethics Committee Ile de France Sud (Registration number: 07-004).

### Domestic animal samples and bioassay in mice

*T. gondii* isolated from animals originating from eight different areas in Gabon (Figure S1) between February 2007, and December 2007, were analyzed. Samples were collected from the households of four main areas: Libreville (latitude: 0° 23′ North, longitude: 9° 27′ East), Franceville (latitude: 1° 37′ South, longitude: 13° 34′ East), Makokou (latitude: 0° 33′ North, longitude: 12° 50′ East), and Dienga (latitude: 1° 52′ South, longitude: 12° 43′ East), a small rural village with a high prevalence of *T. gondii* infection [21]. Contrary to Dienga, the three localities Libreville, Franceville and Makokou are considered as urban environments. Occasionally, samples were obtained from Bakoumba (latitude: 1° 42′ South, longitude: 12° 53′ East), La Lopé (latitude: 0° 05′South, longitude: 11° 36′ East), Léconi (latitude: 1° 35′South, longitude: 14° 15′ East), and around Mougoundou, a Congolese village near the border with Gabon, 10 km south of Dienga, (latitude: 1° 57′ South, longitude: 12° 39′ East). These areas were located 10 to 570 km apart from each other.

For a seroprevalence study, 425 animals were screened for *T. gondii* antibodies at 1∶20, 1∶40, 1∶400 and 1∶800 dilutions using a modified agglutination test (MAT) technique. A total of 267 domestic animals (>1-year old) had positive *T. gondii* antibody titres >1∶20.

According to the availability of animals at each site and for homogeneity of sampling in different locations, 72 seropositive animals were selected for bioassay in mice. For Dienga, 19 samples were taken from free-range chickens, and 19 from other domestic animals to give an overview of the strains present in the village: 12 goats, six sheep, and one domestic cat. Thirty-four free-range chickens were obtained in the seven other geographically distant locations: 17 were collected in Libreville, six in Franceville, seven around Makokou, and one in Bakoumba, La Lopé, Léconi, and Mougoundou. The animals were purchased and brought alive to the International Medical Research Centre of Franceville (CIRMF) where they were bled and euthanized. 0.5–1.5 ml of serum was stored at +4°C until use.

Adult Swiss mice (*Mus musculus*) (Charles River France, L'Arbresle, France) three to seven weeks of age were used in this study. They were individually housed in level two bio safety facilities at CIRMF. Brain and cardiac muscle tissue from seropositive domestic animals (4.2–50 g) were homogenized in 125–250 ml 0.9% NaCl containing 0.4% trypsin and 40 µg/ml gentamycin and incubated for 90 min at 37°C. The suspensions were filtered through fine mesh gauze, washed three times by centrifugation for 10 min at 433 g. The pellets were then resuspended in 0.9% NaCl before inoculation (700 µl i.p.) into mice (3–6 per group). A 200 µl aliquot from this suspension was also used for DNA extraction and quantification of *T. gondii* by real-time quantitative PCR assay targeting the 200- to 300-fold repetitive 529 bp DNA fragment (GenBank accession number AF146527) [22].

Inoculated mice were monitored daily for clinical signs of acute toxoplasmosis; i.e. roughcast hairs, ascites, tottering gait, hunched appearance with evidence of early emaciation and dehydration. In case of clinical signs, the presence of tachyzoites was examined in peritoneal exudates by microscopy. Surviving mice were tested for *T. gondii* antibodies at four weeks by MAT starting at a 1∶20 dilution. All surviving mice were euthanized at four to six weeks post-inoculation. Microscopic examination was performed for the detection of cysts in brain. Depending on the virulence of the isolate, ascites with tachyzoite forms and/or brain tissue suspensions with cyst forms were collected and aliquots (200 µl) for DNA extraction stored at −20°C. Live parasites were cryopreserved in liquid nitrogen with RPMI containing 10% FCS and 10% DMSO. All samples were sent to the *T. gondii* Biological Resource Centre (BRC *Toxoplasma*) laboratory of Limoges, for genotyping studies.

### Genotyping of *T. gondii* isolates

Sixty-eight *T. gondii* isolates were obtained by bioassay in mice. DNA from ascitic fluids or brain tissue was extracted using the QIAamp DNA MiniKit (Qiagen, Courtaboeuf, France). Reference strains, obtained from BRC *Toxoplasma*, were studied in parallel with the new isolates: reference strains for Type I (GT1, ENT, and B1), Type II (Me49 and PRU), and Type III (CTG, VEG and NED) and seven other reference strains originating from Africa [an *Africa 2* strain (CCH002-2004-NIA), and an *Africa 1* strain (DPHT)], South America (TgCkBr93, TgCkBr59, TgCkBr40), Caribbean islands (ENVL-2002-MAC), or France (GPHT) [4], [17], [18], [23], [24], [25], [26].

Genotyping was performed using the length polymorphism of 13 multilocus MS markers located on 10 different chromosomes (Table S1), in 2 multiplex PCR assays. The first multiplex assay included 7 MS markers, *TUB2*, *W35*, *TgM-A*, *B18*, *B17*, *M33* [17], [19] and *M48* [20]. Six other MS markers were used in the second multiplex PCR assay: *AA*, *N82*, *N83*, *N60*, *N61* [18], and *M102* [20]. We also sequenced the W35 marker region as described elsewhere [4]. This was done because polymorphism of Type II and III strains does not differ by fragment length but by the nature of the tandem repeats (TC)7(TG)2 for Type II and (TC)6(TG)3 for Type III [19].

Primers for PCR (sequences are shown Table S1) were synthesized by Applied Biosystems, France. For multiplex PCR assays we used the QIAGEN Multiplex PCR kits (Qiagen, France) with 2x QIAGEN Multiplex PCR Master Mix (final concentration 1x), 0.04 µM of each primer, 5.5 µl distilled water and 4 µl DNA in a total volume of 25 µl. Amplifications were carried out in a GeneAmp PCRSystem 2700 thermalcycler (Applied Biosystems, France): 15 min at 95°C, followed by 40 cycles consisting of 94°C for 30 s, 61°C for 3 min, and 72°C for 60 s. The last extension step was at 60°C for 30 min. Electrophoresis of PCR products was carried out on an ABI Prism 3130xl genetic analyzer (Applied Biosystems, France) and data were stored and analyzed with GeneMapper analysis software (version 4.0, Applied Biosystems, France).

### Data analysis

#### Genetic and genotypic diversity, population genetic structure and linkage disequilibrium

Two indexes were used to describe genetic and genotypic diversity. Nei's unbiased genetic diversity *H*_S_ [27] was estimated for the whole population and within geographic subsamples and genotypic diversity was calculated from the number of multilocus genotypes on the total number of individuals (strains). To evaluate the possible impact of genetic exchanges on the population under survey, the population structure was explored by a set of complementary statistical tests. *F*-statistics are the most widely used parameters to assess population structure [28], [29]. For *F*_ST_ value estimations, we used samples from the geographic populations of Dienga, Libreville, Franceville and Makokou. To take into account the Allendorf-Phelps effect due to small sample size, we corrected *F*_ST_ values by subtracting 1/(2*S*) from the *F*_ST_ values, *S* being the mean number of sampled individuals of each pair of colonies [30]. Data were analyzed with the software FSTAT (version 2.9.4; [31]), which computes estimates, and tests their significance using randomization procedure (*n* = 10,000 randomizations was used).

Linkage disequilibrium (*LD*) between pairs of loci (nonrandom association of alleles at different loci) was assessed with a randomization test (genotypes at two loci are associated at random a number of times) performed in FSTAT. The statistic used was the log likelihood ratio G summed over all subpopulations. Because this procedure was repeated on all pairs of loci, we applied the sequential Bonferroni correction [32] to the *p* values (*p* value divided by number of tests). *LD* was calculated for the two largest geographic populations: Libreville and Dienga. A good estimation of the population *LD* for Franceville and Makokou was not possible due to lack of sufficient isolates. The *LD* estimation inside haplogroups in sympatric conditions was calculated only in Dienga (sufficient number of isolates).

#### Clustering analysis

The entire dataset comprising 69 *T. gondii* strains (one more strain than the number of isolates due to a mixed infection) was submitted for clustering analysis using STRUCTURE 2.2 software [33] to explore Gabonese *T. gondii* population structure. For a spatial coherence, reference strains, not originating from Gabon, were not included in this analysis. STRUCTURE uses Bayesian Monte-Carlo Markov Chain sampling to identify the optimal number of clusters *K* for a given multi-locus dataset by minimizing departures from Hardy-Weinberg and linkage equilibrium expectations, without needing to identify population subunits *a priori*. We used 1,000,000 generations, of which the first 100,000 were discarded as burn-in, and applied the admixture model with correlated allele frequencies. We simulated the dataset for *K* = 1 through to *K* = 10 and performed 10 STRUCTURE runs for each value of *K*. We then employed the methods of Evanno and colleagues [34] and Garnier and colleagues [35] to assess the optimal value of *K* (i.e. the optimal number of clusters in the dataset). Simultaneously, STRUCTURE calculates the proportion (*q_ik_*) of each genotype of individual samples that is derived from each of the *K* clusters. Individual samples can have membership in multiple clusters, but membership coefficients (*q_ik_* values) sum to unity across clusters. Clusters, obtained using Bayesian model (STRUCTURE), were also analyzed with *F*-statistic.

To quantify the extent of genetic distance among Gabonese populations and evaluate their position towards the three reference Types and strains from different continents, Neighbor-joining trees were reconstructed from the genetic distances among individual isolates using Populations 1.2.30 (1999, Olivier Langella, CNRS UPR9034, http://bioinformatics.org/~tryphon/populations/). Only reference strains for Type I, II, and III were included in a first individual distance tree and all the reference strains were added in a second divergence tree based on individuals, but showing only the genotypes for a clearer graphic representation. As recommended by de Meeus and colleagues [36] and Takezaki and colleagues [37], trees were reconstructed using the Cavalli-Sforza and Edwards chord-distance estimator [38]. This analysis was repeated for 1000 bootstrap replicates in which loci were sampled with replacement. Unrooted trees were obtained with R 2.10.1 software [39].

#### Mouse virulence

To determine the association between multilocus genotypes and virulence phenotypes in mice, as already described by Pena and colleagues [5], parasite virulence was defined at the time of parasite isolation by mouse mortality within four weeks of infection (Table S2). Unlike Pena and colleagues [5], we relied on haplogroups instead of genotypes to increase the number of mice for calculation of cumulative mortality and added the estimate of the inoculum dose by real-time PCR to evaluate a possible dose effect on virulence.

The following response variables were analyzed: survival time, and presence or absence of ascites. Two explicative variables were also tested: haplogroups (three modalities: *Africa 1*, *Africa 3*, and *Type III*) and dose of parasites present in inoculums classified in three classes (three modalities: <100, [100-1000[, ≥1000 parasites, according to usual definition of *T. gondii* virulence in mouse model; [10]). Two strains (GAB3-2007-GAL-DOM5A and GAB3-2007-GAL-DOM5B) present in mixed infection were excluded from the analysis. Another isolate (GAB1-2007-GAL-DOM20) was also excluded due to early sacrifice after inoculation.

A Cox proportional-hazard regression analysis was performed to explain the relation between survival time and the two factors, haplogroup and dose, while the Kaplan–Meier method was used to estimate the survival curves of the different haplogroups.

To refine the relationship between haplogroup and virulence in mice, haplogroups were also characterized using another phenotypic trait of virulence, the presence of ascites. Association between presence of ascites and haplogroup membership of the isolates adjusted for dose effect was evaluated by a multivariate logistic regression analysis. Statistical analyses were performed using SAS software (version 9.1.3; SAS Institute) and level of significance was 0.05.

## Results

### Allelic polymorphism and genetic diversity

Three MS markers showed low allelic polymorphism: one allele for *B18*, *M33* and *N82*, two alleles for *TUB2*, *TgM-A*, *B17* and *M102*, three for *W35* and four for *N60*. Higher allelic polymorphism was found for the other MS markers, particularly *N61* and *AA* with nine alleles (Table S2). Compared to data obtained from other continents, there were no novel variant alleles on the 13 MS markers among these isolates. The mean genetic diversity (*H*_S_) for the whole Gabonese population was 0.29. HS values were 0.12, 0.27, 0.26 and 0.27 for the populations of Dienga, Libreville, Franceville and Makokou, respectively.

Overall, a total of 27 different genotypes based on 13 MS markers was found in the population of 69 Gabonese animal strains (Table S2). Fourteen of them differed from another genotype by only one MS marker. Twelve genotypes comprised two or more isolates, while 15 genotypes corresponded to a single isolate. Each genotype was confined to one geographic area, if we considered Dienga and Mougoundou (located at 10 km apart), which shared the same genotype (#12), as one unique area. In Libreville or Dienga, eight genotypes were found, while four genotypes were found in Franceville or Makokou. The genotypic diversity was 0.39 (27/69).

A mixed infection found in one isolate (GAB3-2007-GAL-DOM5) was identified by the presence of two alleles at six loci: *W35* (248 and 242), *TgM*-*A* (205 and 207), *M48* (227 and 229), *N60* (147 and 142), *N83* (131 and 135), and *N61* (128 and 134) (Table S2). Remarkably, two *T. gondii* strains, A and B, were identified based on differential virulence in the mouse bioassay. Mice infected with strain “A” produced ascites with tachyzoites, while strain “B” produced only brain tissue cysts. *T. gondii* genotyping of ascites identified a lone genotype #4. Genotyping of brain tissue cysts detected mixed genotypes. Knowing the MS profile of genotype #4, genotype #9 was deduced from the mixed genotypes.

### Clustering analysis

#### STRUCTURE analysis

**Figure 1:**
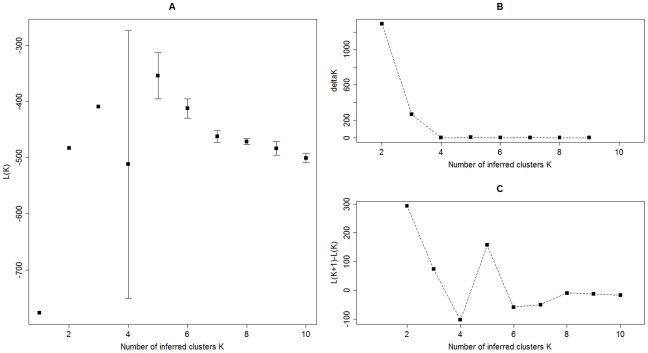
Determination of the optimal value of K (i.e. the optimal number of clusters in the dataset). A. Mean (± SD) of *Ln P(D)* over 10 STRUCTURE runs for successive *K* values ranging from 1 to 10, B. Variations of *ΔK* as calculated by Evanno and colleagues [34] and C. Variations of L(K+1)-L(K) as calculated by Garnier and colleagues [35] for successive *K* values.

**Figure 2:**
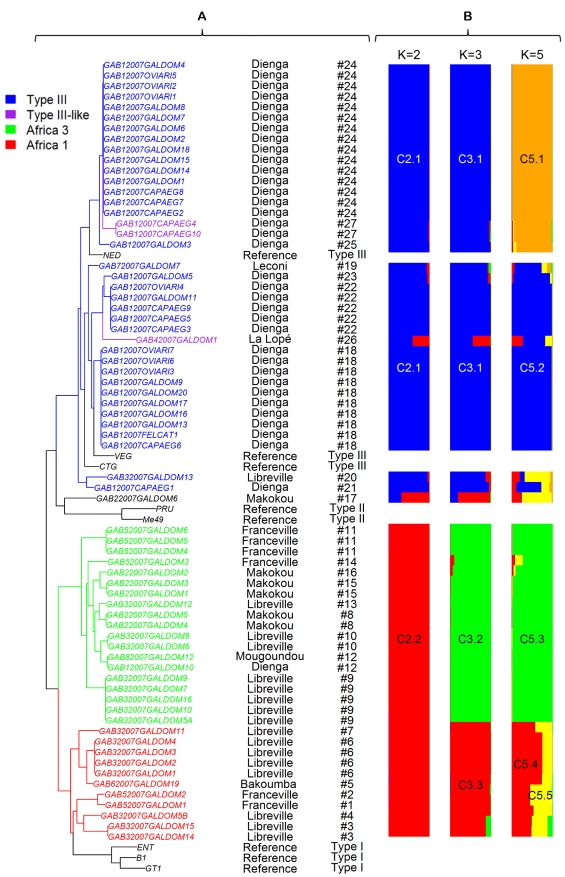
Neighbor-joining tree and clusters as inferred from Cavalli-Sforza distances and by STRUCTURE calculated for 13 microsatellite markers and on all 69 Gabonese *T. gondii* strains. A: Neighbor-joining tree as inferred from Cavalli-Sforza distances on all 69 Gabonese *T. gondii* strains and seven Type reference strains. Midpoint rooting was applied for the Neighbor-joining tree; no outgroup was used. Geographical origin and genotype membership (# genotype number) for each strain are reported. Colors correspond to colors of populations found with STRUCTURE for K = 3 (blue, green and red for respectively cluster C3.1, C3.2 and C3.3 and black for the single isolate strain: GAB4-2007-GAL-DOM1 and for Type reference strains), except for *Type III-like* isolates which are in purple. B: Clusters as inferred by STRUCTURE on all 69 Gabonese *T. gondii* strains. K = 2, K = 3 and K = 5 are indicated by bars in different colors next to the tree. The partition (i.e. relative *q_ik_*) of each of the 69 *T. gondii* strain genotypes within each cluster for the most likely number of inferred clusters K = 2, 3 and 5 is presented.

The results of analysis with STRUCTURE without a priori information on sample location are presented in figures 1 and 2B. The variation of the Ln P(D) values with the number of inferred clusters *K* (Figure 1A) indicated that *K* = 2 is the most likely number of clusters followed by *K* = 3 and *K* = 5. This finding was also confirmed using the Evanno and colleagues [34] (Figure 1B) and Garnier and colleagues [35] (Figure 1C) methods.

Based on clustering solution *K* = 2 (Figure 2B), we found that cluster C2.1 comprised all the isolates (except one: GAB1-GAL-DOM10) from Dienga, while Libreville and Léconi were represented by only one isolate respectively. Two isolates originating from Makokou and La Lopé respectively were admixed (i.e. had a genome nearly equally shared between two clusters). All the other isolates belonged to cluster C2.2 whatever their location.

Clustering solutions *K* = 3 and *K* = 5 are the results of sub-clustering of the more likely solution *K* = 2. From *K* = 2 to *K* = 3, two sub-clusters of C2.2 were found so that the cluster C3.3 was now quasi-exclusively constituted by isolates from both Libreville and Franceville whereas the cluster C3.2 also included Makokou isolates. From *K* = 3 to *K* = 5, the main change in clustering was due to the partition of cluster C3.1 (mainly Dienga strains) in two sub-clusters C5.1 and C5.2, and two admixed strains (GAB3-2007-GAL-DOM13 and GAB1-2007-CAP-AEG1). Cluster C3.3 was divided into two sub-clusters C5.4 and C5.5.

#### Distance analysis

**Figure 3:**
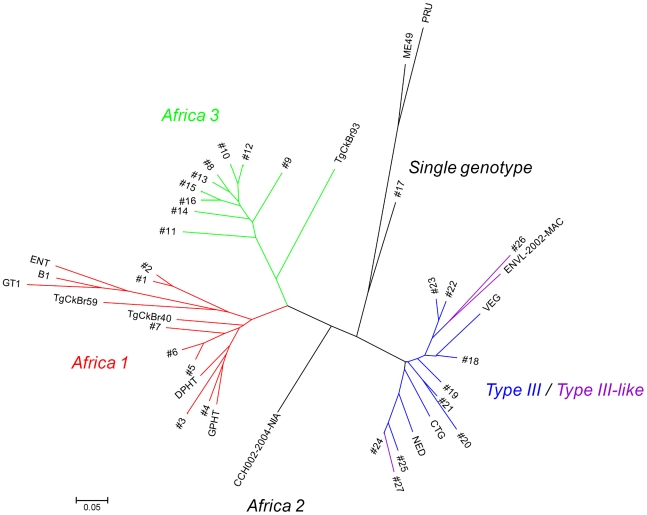
Neighbor-joining tree of genotypes inferred from Cavalli-Sforza distances calculated for the data of 13 microsatellite markers and all 69 Gabonese isolates, including also all the reference strains. [Seven Type reference strains: Type I (GT1, ENT, and B1), Type II (Me49 and PRU), Type III (CTG, VEG and NED), *Africa 2* (CCH002-2004-NIA) (Ajzenberg et al. 2009), and *Africa 1* (DPHT) (Ajzenberg et al., 2009), and seven reference isolates originating from different countries: France (GPHT), Brazil (TgCkBr93, TgCkBr59, TgCkBr40), and Caribbean islands (ENVL-2002-MAC)]. Note: Midpoint rooting was applied for the Neighbor-joining tree; no outgroup was used. Colors correspond to colors of populations found with STRUCTURE for K = 3 (blue, green and red for respectively cluster C3.1, C3.2 and C3.3 and black for the single isolate strain: GAB4-2007-GAL-DOM1 and for Type reference strains), except for *Type III-like* isolates which are in purple.

In addition to STRUCTURE analysis, a genetic distance-based approach was also used to define the population structure of Gabonese *T. gondii* strains and make comparison with other reference strains (Table S2, Figure 2A, 3). Remarkably, the distance trees also identified the two major clusters defined by STRUCTURE showing the robustness of this clustering. However, they were not supported by significant bootstrap values (data not shown). Genetic trees based on Neighbor-joining showed that the 27 genotypes (Figure 3) could be also clustered in three main groups: a group clustering *Africa 1* haplogroup and *Type I*, a group clustering *Type III* and *Type III-like* strains, and haplogroup *Africa* 3. These groups correspond to the hypothesis *K* = 3 of STRUCTURE with subclustering in two clusters of C2.2, which also confirms the robustness of this clustering. One single genotype (GAB2-2007-GAL-DOM6) did not fit into these main groups. The *Africa 1*, *Type III*, and *Africa* 3 haplogroups included 11, 35, and 19 strains respectively (Table 1). The three isolates from Type III-like group did not form a homogenous group inside Type III/III-like group.

**Table 1:** Mouse virulence among haplogroups of *T. gondii* isolates.

Factors		*T. gondii* haplogroups			
	*Africa 1*	*Type III*	*Type III-like*	*Africa 3*	*Single genotype*
Number of strains	11^a^	35	3	19^b^	1
Number of mice infected	33^a^	124	6	57^b^	3
Inoculum dose classed by strains: <100	0 (0)	19 (54.3)	2 (66.7)	2 (11.1)	0 (0)
[100-1000[	5 (50.0)	12 (34.3)	1 (33.3)	5 (27.8)	0 (0)
≥1000	5 (50.0)	4 (11.4)	0 (0)	11 (61.1)	1 (100)
Presence of ascites by number of mice	27 (90.0)	7 (5.8)	0 (0)	28 (51.8)	0 (0)
Number of mice dead at 4 weeks p.i. (cumulative mortality)	27 (90.0)	10 (8.3)^c^	1 (15.0)	52 (96.3)	0 (0)

Note. Data are no. (%) of subjects.

a These numbers include the strain GAB3-2007-GAL-DOM5B which was excluded from the following calculation and corresponded to three mice in the study.

b These numbers include the strain GAB3-2007-GAL-DOM5A which was excluded from the following calculation and corresponded to three mice in the study.

c These values does not include the strain GAB1-2007-GAL-DOM20 which corresponded to three mice in the study.

With MS markers used for typing strains (TUB2, W35, TgM-A, B18, B17, and *M33*) [17], these haplogroups were characterized by different combinations of classical Type I, II, or III alleles. Our *Type III* isolates were identical with these markers to Type III reference strains. Type III-like strains (genotypes #26 and #27) differed from Type III by a Type I allele for TUB2. With these same MS typing markers, the two other haplogroups and the single isolate were different from the three classical lineages: *Africa 1* strains harbored defined mixtures of I, and III alleles, while *Africa 3* strains and the single genotype were a mix of alleles I, II, and III.

However on the divergence tree analysis, which is based on all the 13 MS markers (Figure 3), *Africa 1* strains and the single genotype clustered with Type I and Type II reference strains, respectively. *Africa 1* also clustered with GPHT, DPHT, TgCkBr59, and TgCkBr40 strains. *Africa 3* clustered with TgCkBr93, and Type III and III-like strains with Type III reference strains. ENVL-2002-MAC strain was closed to one of the *Type III-like* strains (GAB4-2007-GAL-DOM1). The *Africa 2* strain (CCH002-2004-NIA) was apart from all the other strains.

The *Type III/III-like* group (equivalent to cluster C2.1 of STRUCTURE with *K* = 2) included strains from Dienga, and two from Libreville and Léconi. Among Type III isolates from Dienga, a less supported subclustering was observed corresponding to the two sub-clusters C5.1 and C5.2, and the two admixed strains from *K* = 5 of STRUCTURE (Figures 2A, B). Likewise, in Africa 1 haplogroup, as STRUCTURE analysis divided Cluster C3.3 into two sub-clusters C5.4 and C5.5, divergence tree showed the same less supported subclustering.

Inside *Africa 3* haplogroup, the Makokou isolates also formed a homogeneous subgroup (Figure 2A). Isolates from Libreville and Franceville were distributed in *Africa 1* and *3* haplogroups, with less supported subclusters of isolates in each town.

### Population structure analysis

Regarding the geographical populations, all pairwise *F*_ST_ values from Dienga, Makokou, Libreville and Franceville, were significant (*p* = 0.0083) with values ranging between 0.11 and 0.64. In addition, pairwise *F*_ST_ values for the Dienga population *vs* the three urban populations (Makokou, Libreville and Franceville) were higher (0.56–0.64) than pairwise *F*_ST_ values between these three latter populations (0.11–0.19) (Table 2). The two main clusters (*K* = 2) and subclusters (*K* = 3 and *K* = 5) recognized by model-based and distance based analyses were also supported by *F*-statistics. All *F*_ST_ values were >0.22 and significant (*p* **≤**0.05) indicating strong genetic differentiation between these clusters.

**Table 2:** *F*_ST_ estimates of inter-geographical population differentiation for four subpopulations based on microsatellite data.

Populations	Dienga	Makokou	Libreville	Franceville
Dienga	*	*0.0083*	*0.0083*	*0.0083*
Makokou	0.64	*	*0.0083*	*0.0083*
Libreville	0.56	0.11	*	*0.0083*
Franceville	0.64	0.19	0.12	*

Note. Italics indicate p-values generated from 10 000 random permutations leading to a value larger than or equal to that observed. “Allendorfs phelps” effect has been taken into account for each *F*_ST_ value.

Concerning the *LD* calculations for Libreville and Dienga populations, 9 out of the 45 pairs (20%, *n* = 10 polymorphic loci) and 12 out of the 36 pairs (33%, *n* = 9 polymorphic loci) remained in significant linkage disequilibrium, respectively, after sequential Bonferroni correction. This cannot be attributed to close physical linkage between loci, since the 13 loci were distributed among 10 different chromosomes, so that all but three pairs among the 75 involve loci located on different chromosomes (Table S1). These findings indicated strong linkage at a genome-wide scale.

For *Type III* population from Dienga (sympatric conditions), the *LD* was calculated. Five out of 10 pairs (50%, *n* = 5 polymorphic loci, all on different chromosomes) remained in significant linkage disequilibrium, after sequential Bonferroni.

### Analysis of haplogroups and virulence in mice

The characteristics used to define virulence for each isolate are shown in Table S2. Inoculum dose ranged from one to 44 600 parasites in the total volume inoculated. The majority of strains belonging to haplogroups *Africa 1* and *Africa 3* were isolated from tissue samples with higher dose classes (≥1000 parasites or between 100 and 1000 parasites), than *Type III* strains (<100 parasites) (Table S2 and 1). A Fisher exact test found that the three groups were significantly different according to dose classes (*p*<0.0001). Considering these results, all the further analyses were adjusted on dose effect. The single genotype and *Type III-like* genotypes appeared to be weakly pathogenic, the small sample size was not amenable to statistical analysis and, therefore, these strains were excluded from further analyses.

**Figure 4:**
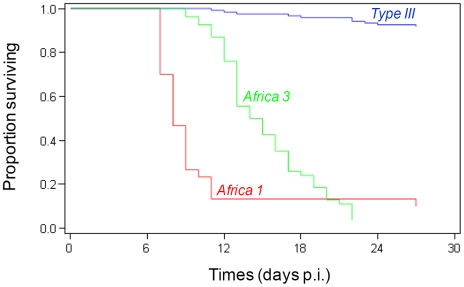
Proportion of surviving mice infected with *T. gondii* haplogroups.

Using the Cox proportional-hazards regression analyses, we assessed the relationships between survival time and the two factors: dose and haplogroup membership of the isolates. The dose factor was slightly significant (*p* = 0.04) in relation with survival time compared to haplogroup membership factor (*p*<0,001) (Table 3). The proportion of mice killed by *Type III* isolates (8.3% at four weeks) indicated a weakly virulent phenotype, whereas *Africa 1* and *3* isolates demonstrated a highly virulent phenotype (mortality greater than 90%) (Table 1). The survival curves showed that mice infected with strains harboring alleles I (*Africa 1* and *3*) had a shorter median survival time (respectively 8 days and 14 days) compared with those infected with *Type III* (median survival time not reached) (Figure 4). The estimation of the hazard-ratio adjusted for quantity of parasites, showed that isolates from the *Africa 1* haplogroup killed mice nearly two times faster than isolates from the *Africa 3* haplogroup (Figure 4, Table 3). According to logistic regression analysis, the presence of ascites adjusted for dose effect, was significantly associated with haplogroups (*p*<0. 001). Ascites occurred in 60 (67.4%) of 89 mice which died before 4 weeks and only for 2 (1.7%) of the 116 surviving mice. No interaction between dose and haplogroup was found in both statistical models.

**Table 3:** *P* values and hazard ratios (95% IC) for mouse survival time estimated from the Cox proportional-hazard regression analysis.

Factor	Hazard Ratio*	IC 95%	P
**Haplogroup effect**			**<0.001**
*Africa 1 vs Type III*	62.2	26.0–148.5	<0.001
*Africa 3 vs Type III*	28.2	12,9–61,2	<0.001
*Africa 1 vs Africa 3*	2.2	1.3–3.7	<0.001

Note: Mouse survival time was studied during 28-days post-inoculation.

*Hazard ratio were adjusted on the number of inoculated parasites.

## Discussion

Compared to Europe and the Americas, the population structure of African *T. gondii* has been underexplored. Previous studies were based on a limited number of isolated African strains and markers used to characterize these strains defined the Type level [14], [15], [16], [17] but not the population structure. The development of MS markers [18], [20], that discriminate within these Types, has for the first time enabled us, to reliably address or define important epidemiological issues such as i) the diversity of strains and groups, ii) the existence of geographical subpopulations, and iii) the extent of gene flow throughout *Toxoplasma* circulation between these subpopulations. The impact of homoplasy with MS markers is well known [40]. In this work it has been minimized by the selection of variable and numerous (*n* = 13) MS markers located, for most of them, on different chromosomes (Table S1). Moreover, this effect was shown as being not a matter of concern in population genetics [40].

This study represents the most comprehensive attempt to document within African diversity in *T. gondii* to date. Nonetheless, some sample sizes remain a limit in population genetic terms, although efforts were made to correct for any confounding effects. Similarly, caution is required given the deviation of *T. gondii* from the assumptions of most standard population genetic models due to clonality.

Our results, with 27 genotypes out of 69 strains, suggest a diverse *T. gondii* population in the Gabon area. However, the mean genetic and genotypic diversities could be considered as moderate, compared to data sets from other continents which were studied with equivalent MS characterization [4], [41]. A very high diversity in *T. gondii* strains was found in the wild environment of the Amazonian rainforest in French Guiana and Surinam [4], [6]. The mean genetic diversity for Gabonese strains was similar to what has been found for 104 French animal isolates from three regions (French BRC for *T. gondii*, personal data). This moderate and similar genetic diversity in the two environments (French and Gabonese animals) could be explained by a comparable degree of anthropization with domestication of cats (definitive host) and intermediate hosts for sampling areas [4]. Allelic polymorphism for each MS marker is comparable to polymorphism observed in other studies using the same markers [1], [41]. The few differences observed for some loci, as the monomorphic version of N82 marker, may be explained by the diverse geographic areas studied. No novel alleles was found in this African sample population.

The Bayesian model for predicting population structure, distance-based analysis methods, and *F*-statistics analysis resolved partitions among the total sample of 69 isolates. All these tests were concordant and clearly demonstrated the existence of three main haplogroups among strains sampled in Gabon, *Type III*, *Africa 1* and *3* haplogroups. In addition to genetic factors, phenotypic factors provided by virulence analysis (survival time and presence of ascites) distinguished these three haplogroups (see below). Except for *Type III*, the two other haplogroups did not correspond to classical Type I and II, This result contrasted with studies of isolates collected from chickens in several African countries showing a predominance of classical lineages in these areas: mainly Type III and some Type II [15], and a majority of Types I and II, with just one Type III in Uganda [14]. The most remarkable difference with our study is the predominance of Type II strains in these studies. This may be explained by the geographic origins of samples. Type II was found predominantly in Uganda, a Central-East African country, whereas other studies [15], [17], and this one used mainly samples from Western and Middle Africa.

The finding of Type III in our Gabonese isolates confirms the widespread distribution of this Type, already described in North and South America, Europe, Africa, and Asia [3], [15], [18], [42], [43]. But in Gabon, except for two strains from Libreville and Léconi, all the Type III strains came from the small village of Dienga. We might have expected the opposite phenomenon (Type III in the large cities of the country) if the global spread was due to recent migration [1]. An explanation may be the limited sampling in urban areas. The location of Type III in a remote rural area of Africa suggests either a recent introduction in this village from an imported animal (the high *LD* argued for a local clonal spread of this lineage) or that Type III is ancestral in Africa. This last hypothesis on the ancestral nature of Type III is in agreement with the hypothesis concerning *ROP18* III previously proposed [44].

Velmurugan and colleagues [15] also found genotypes different from the classical Types II and III. Due to different genotyping markers, it is not possible to compare these non-classical genotypes with those described in the present study. In Lindstrom and colleagues [14], incomplete genotyping for some isolates (only two to four markers for defining Type I) could have misidentified non classical isolates or recombinant genotypes. Among the non-classical haplogroups, the *Africa 1* was also found in patients originating from other West and Central African countries [17]. It has been collected at different times in various and distant areas, from Senegal to Uganda through Gabon. Another haplogroup described for the first time in this study, *Africa 3* was largely distributed in Gabon and represents another major haplogroup in Africa. The *Africa 2* haplogroup, described by Ajzenberg and colleagues [17], was not found in Gabon.

The question of endemicity of the African haplogroups described in this study must be addressed. In different papers, other strains (GPHT, FOU) genotyped with microsatellite markers as *Africa 1* [4], [17] clustered with one of the Brazilian Type (*BrI*) defined by PCR-RFLP markers [5] or with other Brazilian strains in haplogroup 6 defined sequencing of introns [7]. We confirmed these observations by using these strains and other strains from South or Central America, or from Africa as reference strains in our genotypic divergence tree (Figure 3). These strains clustered either with *Africa 1* or *3* haplogroups, or with one of the *Type III-like* strains. These findings show that these African and American strains share a common ancestor and support the hypothesis which suggested the possibility of *T. gondii* migration during the transatlantic slave trade during the 18th and 19th centuries [1],[7]. The model for dissemination of *T. gondii* strains proposed by Khan and colleagues [7] does not take into account African continent. The widespread distribution of these major haplogroups, together with the propagation considered as predominantly clonal for *T. gondii* in a domestic environment [4], strongly suggest that *Africa 1*, and *3* strains may correspond to new major clonal lineages. More sampling across the world would be needed to confirm this hypothesis.

Whether these additional haplogroups for Africa represent minor variations of Type I, II, and III, or recombinant strains of these three lineages remained to be determined. A deeper sequencing as performed by Lindström Bontell and colleagues [16] has demonstrated the existence of one natural Type II and III recombinant strain inside the previous Uganda isolates data set. Such a sequencing would be needed for strains of our haplogroups. However, even if sequencing demonstrated such recombination, it would be a successful recombinant, widespread over continents, representing a lineage with enhanced fitness, as shown for Types II, I and III [45]. Type I was described as one major lineage. However, considering the literature on multilocus typing of *Toxoplasma* strains, it was rarely encountered in nature [46]. *Africa 1* strains clustered with Type I in divergence trees (Figures 2A, 3). Considering the rarity of Type I and the genotypic diversity within *Africa 1*, one could evoke the possibility of Type I being a divergent strain from *Africa 1*. Regarding the haplogroups *Africa 3* as the *Africa 2*, the question remains.

In our study, we demonstrated a clear relationship between haplogroups and mouse-virulence: isolates of haplogroups with Type I alleles (*Africa 1* and *3*) were significantly associated with presence of ascites and mortality in infected mice, while Type III isolates were associated with survival. *Africa 1* haplogroup was associated with a shorter survival time than *Africa 3* haplogroup (Figure 4). Even if this relationship between haplogroup and virulence at isolation, independent of a dose-effect, was clearly shown using statistical analysis, this should be confirmed by an experimental study in controlled conditions. The higher proportion of elevated doses in inoculums of *Africa 1* and *3* haplogroups compared to Type III, independently of host species (chicken, sheep, goats and cat), was found significant, indicating that parasite burden could be higher in *Africa 1* and *3* naturally infected animals.

Although these data demonstrate different intrinsic properties of the different strains, the expression of this virulence in a given host species is a more complex trait which depends on several host and parasite characteristics [2]. Pathogenicity in humans cannot be deduced from virulence in the mouse model. However, several studies argued for a role of genotypes in the clinical expression of human toxoplasmosis [47], [48], [49], [50], [51]. Notably, a higher proportion of ocular disease was found in South America, associated with certain non-classical genotypes [12], [52]. In Africa, the prevalence of toxoplasmosis in uveitis may be high. It has been estimated up to 43% in Sierra Leone [53]. A 100-fold higher incidence of ocular toxoplasmosis was observed in patients born in West Africa compared to patients born in Britain [54]. The similarity demonstrated in this study between genotypes found in Africa and in South or Central America should encourage further studies in Africa associating clinical data and genotype analysis.

**Figure 5:**
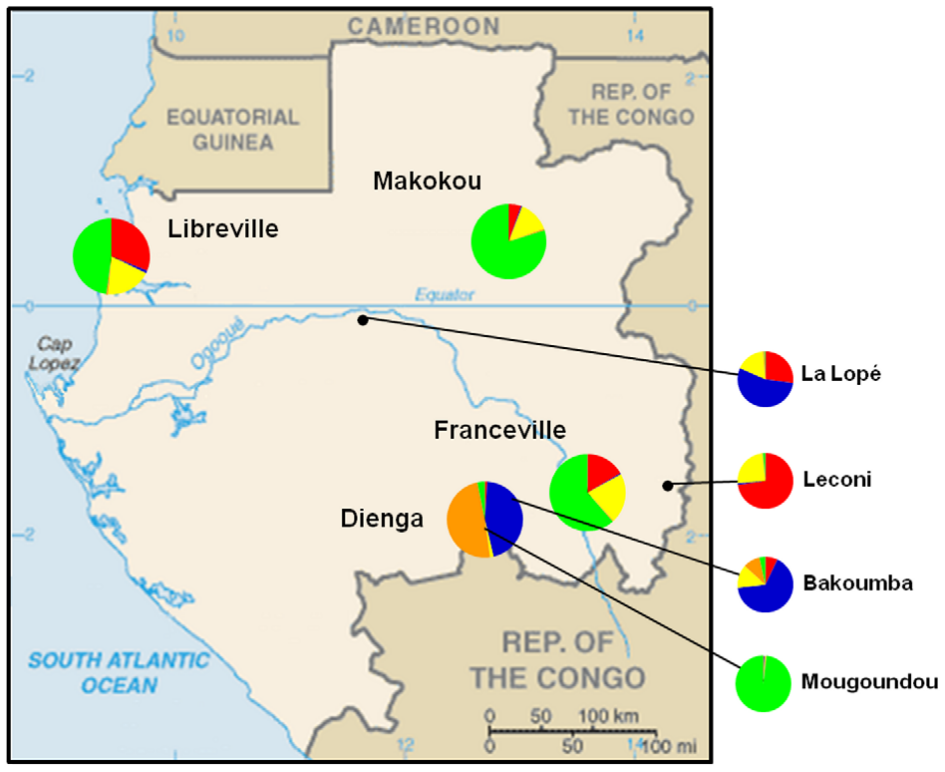
Proportion of membership of each geographic population in each of the 5 clusters (STRUCTURE, K = 5). Note: Adapted from the Central Intelligence Agency Web site [55]. Colors correspond to the populations in Figure 2.

MS analysis permits population structure study on a large as well as local scale. In our study, considering the large distances between areas (from 105 to 590 km apart) and the sampling method, geographic subdivision was expected. The significant genetic differentiation between the populations of Dienga, Libreville, Franceville and Makokou sustains this geographic isolation (Wahlund effect). But isolation by distance cannot explain all genetic differences. Whereas distances between Franceville and Libreville, and Makokou were far more important than the distance between Dienga and Franceville (Figure S1), strains from Franceville are genetically closer to Libreville and Makokou strains than to Dienga strains (Figure 5). Some genotype circulations which would lead to gene flow between these urban populations throughout the history was suggested by i) the higher *F*_ST_ values between Dienga *vs* urban populations than between the three urban populations, ii) the clustering of urban populations (K = 2) by STRUCTURE, iii) their structure similarity (K = 3 and K = 5), and iv) more generally, low bootstrap values in distance genetic analysis (Table 2, Figures 2, 4). It may be explained by economic and human exchanges between the three towns. The trade of animals, food and pets, together with rodents, could be a migration opportunity for *T. gondii* isolates favoring genetic exchanges between isolates of the large cities. Conversely, Dienga is a village with very few trade exchanges with the other locations, which may explain the divergence of this *T. gondii* population. Intensive anthropization and urbanization may have an impact on the circulation of *T. gondii* strains in Africa.

This detailed population genetics study of *T. gondii* is an original process concerning *T. gondii* epidemiology. It demonstrated in an African country the existence of a genetic heterogeneity at a country scale with new major haplogroups and a substantial population structure at a microgeographic scale. The approach used here needs to be applied to strains of *T. gondii* from other African countries and other continents to ascertain these population observations and compare the possible differences of structuring. The geographical genetic structure inside a same country indicates that further epidemiological and clinical studies should integrate different scales (country, districts…) and environment (urban or rural areas, anthropized or wild environment).

## Supporting Information

Aternative Language Abstract S1 Translation of the Abstract into French by Mercier et al.
(0.02 MB DOC)

Figure S1 Map of Gabon with locations of sampling. Note: Adapted from the Central Intelligence Agency Web site [52].
(1.11 MB TIF)

Table S1 Microsatellite markers and PCR primers used for the multiplex PCR assays.
(0.05 MB DOC)

Table S2 Multilocus microsatellite (MS) genotyping and mouse virulence of *Toxoplasma* isolated in domestic animals from Gabon.
(0.31 MB DOC)
