# Supplementary material for: Additional Haplogroups of Toxoplasma gondii out of Africa: Population Structure and Mouse-Virulence of Strains from Gabon
Source: PLoS Negl Trop Dis. 2010 Nov 2;4(11):e876. doi: 10.1371/journal.pntd.0000876 (PMC2970538; doi:10.1371/journal.pntd.0000876)
Supplement: Aternative Language Abstract S1 — Translation of the Abstract into French by Mercier et al. (0.02 MB DOC) [file pntd.0000876.s001.doc]

**Contexte :** *Toxoplasma gondii* est répandu dans le monde entier, mais la répartition de ses génotypes ainsi que l'expression clinique de la toxoplasmose humaine varie selon les continents. Plusieurs études en Europe, en Amérique du Nord et du Sud plaident pour un rôle des génotypes dans l'expression clinique de la toxoplasmose humaine. Les données génétiques concernant les isolats de *T. gondii* en provenance d'Afrique sont rares et n’ont pas permis l’étude de la structure des populations de ce parasite. L’ajout de nouvelles données analysées en termes de génétique des populations permettrait une meilleure compréhension de la distribution, de la circulation et de la transmission de *T. gondii* sur ce continent.

**Méthodologie / Résultats :** L'isolement et le génotypage de souches de *T. gondii* ont été réalisés à partir de prélèvement d’animaux séropositifs provenant de zones urbaines et rurales du Gabon. Soixante-huit isolats, y compris une infection mixte (69 souches), ont été obtenus par bio-essais chez la souris. Le génotypage a été effectué en utilisant le polymorphisme de longueur de 13 marqueurs microsatellites répartis sur 10 chromosomes différents. Les résultats ont été analysés en termes de structure de la population à l’aide de modèle statistique Bayésien, d’arbres de distance (Neighbor-joining), de l’étude des *F*_ST_ et du déséquilibre de liaison. Une diversité génétique modérée a été décrite. Trois haplogroupes et un génotype unique regroupent 27 génotypes. La majorité des souches appartenaient à un seul et même haplogroupe : le Type III dont la répartition semble mondiale. Les souches restantes ont été réparties entre les deux autres haplogroupes (*Africa 1* et *3*) et un génotype unique. La virulence chez la souris de ces souches a été étudiée à l'isolement et des différences significatives ont été obtenues entre les haplogroupes, l’haplogroupe *Africa 1* étant le plus virulent.

**Discussion / Conclusion :** les haplogroupes *Africa 1* et *3* ont été proposés comme étant de nouveaux haplogroupes majeurs de *T. gondii.* Un lien possible avec les souches circulant en Amérique Centrale et du Sud est discuté. L’analyse de la structure de la population a mis en évidence une propagation locale du type III dans une zone rurale et une circulation accrue du parasite entre les principales villes du pays. Cette circulation, favorisée par l'activité humaine pourrait conduire à des échanges génétiques. Pour la première fois, grâce au fort pouvoir discriminant des marqueurs microsatellites, les principales questions épidémiologiques ont pu être testées sur une population conséquente d’isolats de *T. gondii* d’Afrique de l'Ouest. Une base pour de nouvelles investigations épidémiologiques et cliniques a ainsi été créée.
