# Supplementary figures and images for: Additional Haplogroups of Toxoplasma gondii out of Africa: Population Structure and Mouse-Virulence of Strains from Gabon
Source: PLoS Negl Trop Dis. 2010 Nov 2;4(11):e876. doi: 10.1371/journal.pntd.0000876 (PMC2970538; doi:10.1371/journal.pntd.0000876)

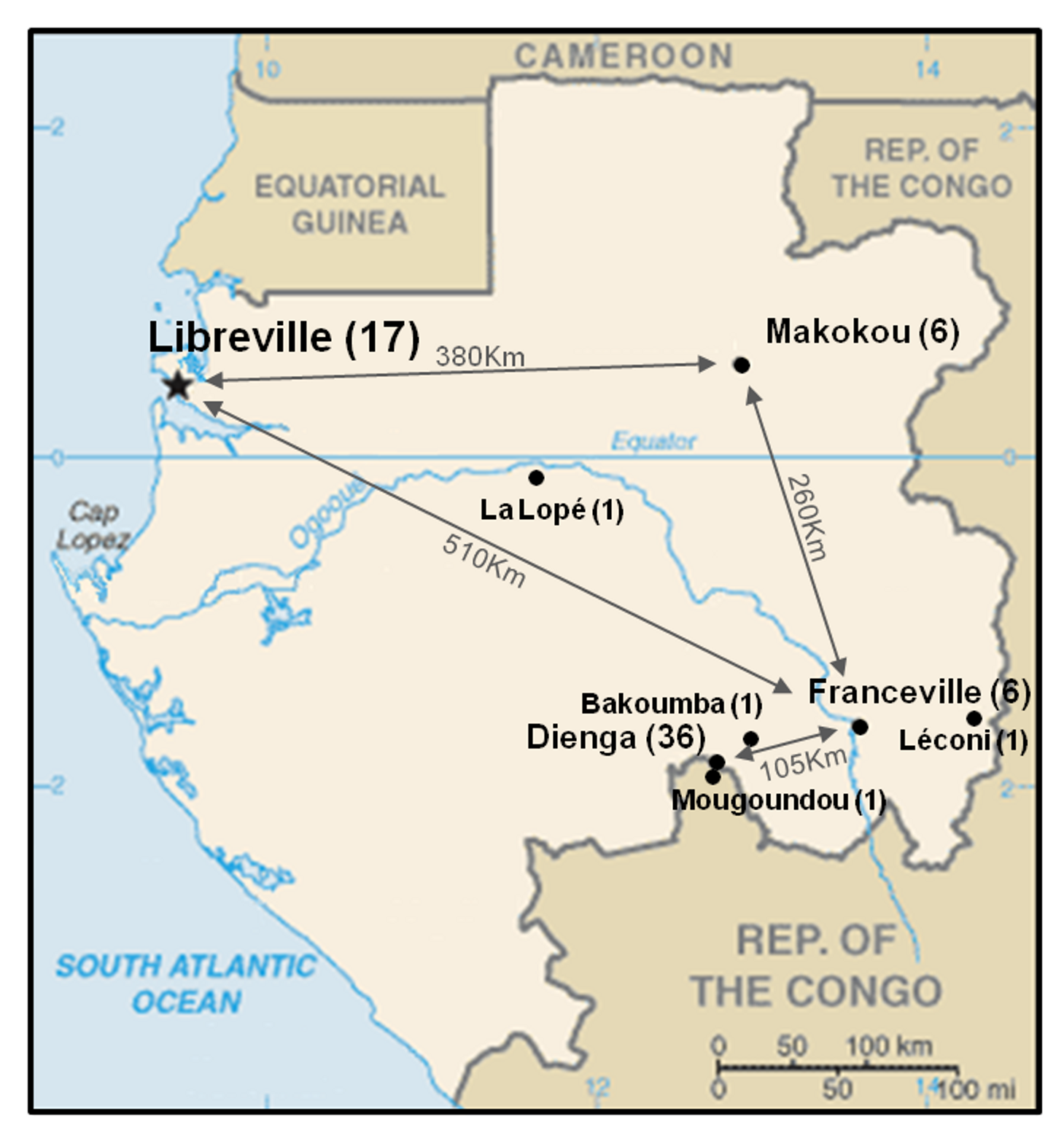

Supplement: Figure S1 — Map of Gabon with locations of sampling. Note: Adapted from the Central Intelligence Agency Web site [52]. (1.11 MB TIF) [file pntd.0000876.s002.tif]
