# Supplementary material for: Additional Haplogroups of Toxoplasma gondii out of Africa: Population Structure and Mouse-Virulence of Strains from Gabon
Source: PLoS Negl Trop Dis. 2010 Nov 2;4(11):e876. doi: 10.1371/journal.pntd.0000876 (PMC2970538; doi:10.1371/journal.pntd.0000876)
Supplement: Table S1 — Microsatellite markers and PCR primers used for the multiplex PCR assays. (0.05 MB DOC) [file pntd.0000876.s003.doc]

Table S1. Microsatellite markers and PCR primers used for the multiplex PCR assays.

| **Marker** | **Chromosome** | **Number of alleles** | | **Repeat type^a^** | **Forward/Reverse Primer (5’-3’)^b^** |
| --- | --- | --- | --- | --- | --- |
| *TUB2* | IX | 2 | (TG)_n_ | | 6-FAM-gTCCgggTgTTCCTACAAAA |
|  |  |  |  | | TTggCCAAAgACgAAgTTgT |
| *W35* | II | 3 | (CT)_n_ | | 6-FAM-ggTTCACTggATCTTCTCCAA |
|  |  |  |  | | AATgAACgTCgCTTgTTTCC |
| *TgM-A* | X | 2 | (TG)_n_ | | HEX-ggCgTCgACATgAgTTTCTC |
|  |  |  |  | | TgggCATgTAAATgTAgAgATg |
| *B18* | VII a | 1 | (CA)_n_ | | 6-FAM-TggTCTTCACCCTTTCATCC |
|  |  |  |  | | AgggATAAgTTTCTTCACAACgA |
| *B17* | XII | 2 | (TC)_n_ | | HEX-AACAgACACCCgATgCCTAC |
|  |  |  |  | | ggCAACAggAggTAgAggAg |
| *M33* | IV | 1 | (ga)_n_ | | NED- TACGCTTCGCATTGTACCAG |
|  |  |  |  | | TCTTTTCTCCCCTTCGCTCT |
| *N82* | XII | 1 | (TA)_n_ | | 6-FAM-TGCGTGCTTGTCAGAGTTC |
|  |  |  |  | | GCGTCCTTGACATGCACAT |
| N83 | X | 6 | (TA)_n_ | | HEX-CACAACGACACCGCTATC |
|  |  |  |  | | CTCTCTATACACAGACCGATTGG |
| *N60* | I b | 4 | (TA)_n_ | | 6-FAM-GAATCGTCGAGGTGCTATCC |
|  |  |  |  | | AACGGTTGACCTGTGGCGAGT |
| *M48* | I a | 7 | (ta)_n_ | | 6-FAM-AACATGTCGCGTAAGATTCG |
|  |  |  |  | | CTCTTCACTGAGCGCCTTTC |
| *N61* | VII b | 9 | (TA)_n_ | | NED-CCGTATCACCAGATCATGTT |
|  |  |  |  | | CTCTCACCTGATGTTGATGTAA |
| *M102* | VII a | 2 | **(**ta)_n_N_8_(ta)_n_N_2_(ta)_n_N(ta)_n_N_2_(ta)_n_N_12_(ta)_n_# | | NED-CAGTCCAGGCATACCTCACC  CAATCCCAAAATCCCAAACC |
| *AA* | Viii | 9 | (TA)_n_ | | NED-GATGTCCGGTCAATTTTGCT  GACGGGAAGGACAGAAACAC |

^a^ Refers to repeat type (e.g. di-nucleotide). #N=GTGGAGAGGGAAGGAAACGATGCAG AGAGAGACAGAGAGCAAGA (44 bp).

^b^ In each pair, the forward primer was 5’-end labelled with fluorescein: 6-carboxyfluorescein (6-FAM) for *TUB2, W35, B18, N82, N60,* and *M48*; hexachlorofluorescein (HEX) for *TgM-A*, *B17,* and *N83*; 2,7',8'-benzo-5'-fluoro-2',4,7-trichloro-5-carboxyfluorescein (NED) for M33, N61, and M102.
