# Supplementary material for: Additional Haplogroups of Toxoplasma gondii out of Africa: Population Structure and Mouse-Virulence of Strains from Gabon
Source: PLoS Negl Trop Dis. 2010 Nov 2;4(11):e876. doi: 10.1371/journal.pntd.0000876 (PMC2970538; doi:10.1371/journal.pntd.0000876)
Supplement: Table S2 — Multilocus microsatellite (MS) genotyping and mouse virulence of Toxoplasma isolated in domestic animals from Gabon. (0.31 MB DOC) [file pntd.0000876.s004.doc]

**Table S2. Multilocus microsatellite (MS) genotyping and mouse virulence of *Toxoplasma*** isolated in domestic animals from Gabon.

| *Isolate ID^a^* | *Host sampled* | *Location* | *Quantification^b^* | *Virulence* |  |  |  | *MS genotype^d^* | | | | | | | | | | | | | *Genotypes* | Haplogroups |
| --- | --- | --- | --- | --- | --- | --- | --- | --- | --- | --- | --- | --- | --- | --- | --- | --- | --- | --- | --- | --- | --- | --- |
|  |  |  |  | *Number of mice with ascites*  *(No./No. Infected)* | *% Mortality*  *in mice (No. died/No. Infected)* | *Day of death post inoculation* |  | TUB2 | W35^e^ | TgM-A | B17 | B18 | M33 | M48 | M102 | N60 | N61 | N82 | N83 | AA |  |  |
|  |  |  |  |  |  |  |  |  |  |  |  |  |  |  |  |  |  |  |  |  |  |  |
| GT1 | Goat | USA |  |  |  |  |  | 291 (1) | 248 (1) | 209 (1) | 342 (1) | 160 (1 or 3) | 169 (1 or 2) | 209 | 168 | 145 | 126 | 119 | 131 | 265 |  | Type I |
| ENT | Human | France |  |  |  |  |  | - | - | - | - | - | - | 209 | 166 | 145 | 126 | 121 | 133 | 267 |  | Type I |
| B1 | Cow | Azores |  |  |  |  |  | - | - | - | - | - | - | 209 | 166 | 147 | 126 | 119 | 131 | 273 |  | Type I |
| Me49 | Sheep | USA |  |  |  |  |  | 289 (2 or 3) | 242 (2) | 207 (2) | 336 (2 or 3) | 158 (2) | 169 (1 or 2) | 215 | 174 | 142 | 130 | 111 | 135 | 265 |  | Type II |
| PRU | Human | France |  |  |  |  |  | - | - | - | - | - | - | 209 | 176 | 142 | 160 | 117 | 135 | 265 |  | Type II |
| CTG | Cat | USA |  |  |  |  |  | 289 (2 or 3) | 242 (3) | 205 (3) | 336 (2 or 3) | 160 (1 or 3) | 165(3) | 215 | 190 | 147 | 128 | 111 | 137 | 269 |  | Type III |
| NED | Human | France |  |  |  |  |  | - | - | - | - | - | - | 209 | 190 | 147 | 130 | 111 | 137 | 267 |  | Type III |
| VEG | Human | USA |  |  |  |  |  | - | - | - | - | - | - | 213 | 188 | 153 | 128 | 111 | 137 | 267 |  | Type III |
| CCH002-2004-NIA | Human | Senegal |  |  |  |  |  | 289 (2 or 3) | 248 (1) | 205 (3) | 336 (2 or 3) | 160 (1 or 3) | 165(3) | 225 | 166 | 145 | 126 | 111 | 133 | 273 |  | *Africa 2* |
| ENVL-2002-MAC | Human | Barbados |  |  |  |  |  | 291 (1) | 242 (2) | 205 (3) | 336 (2 or 3) | 160 (1 or 3) | 165(3) | 213 | 190 | 142 | 126 | 111 | 137 | 277 |  | *Type III like* |
| GPHT | Human | France |  |  |  |  |  | 291 (1) | 248 (1) | 205 (3) | 342 (1) | 160 (1 or 3) | 165(3) | 229 | 166 | 147 | 130 | 111 | 131 | 283 |  | *Africa 1* |
| DPHT | Human | Guinea |  |  |  |  |  |  |  |  |  |  |  | 225 | 166 | 147 | 128 | 111 | 131 | 271 |  | *Africa 1* |
| TgCkBr59 | Chicken | Brazil |  |  |  |  |  | - | - | - | - | - | - | 227 | 166 | 145 | 132 | 105 | 131 | 261 |  | *Africa 1* |
| TgCkBr40 | Chicken | Brazil |  |  |  |  |  | - | - | - | - | - | - | 235 | 166 | 147 | 128 | 111 | 133 | 269 |  | *Africa 1* |
| TgCkBr93 | Chicken | Brazil |  |  |  |  |  | 291 (1) | 242 (3) | 207 (2) | 342 (1) | 160 (1 or 3) | 165(3) | 235 | 174 | 140 | 130 | 111 | 133 | 265 |  | *Africa 3* |
|  |  |  |  |  |  |  |  |  |  |  |  |  |  |  |  |  |  |  |  |  |  |  |
| GAB5-2007-GAL-DOM1 | Chicken | Franceville | [100-1000[ | 3/3 | 100 (3/3) | 9 |  | 291 (1) | 248 (1) | 205 (3) | 342 (1) | 160 (1 or 3) | 165(3) | 231 | 166 | 149 | 126 | 111 | 131 | 277 | #1 | *Africa 1* |
| GAB5-2007-GAL-DOM2 | Chicken | Franceville | [100-1000[ | 3/3 | 100 (3/3) | 8-11 |  | - | - | - | - | - | - | 231 | 166 | 149 | 126 | 111 | 131 | 275 | #2 | *Africa 1* |
| GAB3-2007-GAL-DOM14 | Chicken | Libreville | ≥1000 | 0/3 | 0 (0/3) | Survived |  | - | - | - | - | - | - | 229 | 166 | 142 | 134 | 111 | 131 | 271 | #3 | *Africa 1* |
| GAB3-2007-GAL-DOM15 | Chicken | Libreville | ≥1000 | 3/3 | 100 (3/3) | 9-27 |  | - | - | - | - | - | - | - | - | - | - | - | - | - | #3 | *Africa 1* |
| GAB3-2007-GAL-DOM5 B | Chicken | Libreville | NA | 3/3 | NA | NA |  | - | - | - | - | - | - | 227 | 166 | 147 | 130 | 111 | 131 | 271 | #4 | *Africa 1* |
| GAB6-2007-GAL-DOM19 | Chicken | Bakoumba | ≥1000 | 3/3 | 100 (3/3) | 8-9 |  | - | - | - | - | - | - | 223 | 166 | 147 | 128 | 111 | 131 | 273 | #5 | *Africa 1* |
| GAB3-2007-GAL-DOM1 | Chicken | Libreville | [100-1000[ | 3/3 | 100 (3/3) | 7 |  | - | - | - | - | - | - | 223 | 166 | 147 | 128 | 111 | 131 | 269 | #6 | *Africa 1* |
| GAB3-2007-GAL-DOM2 | Chicken | Libreville | ≥1000 | 3/3 | 100 (3/3) | 7 |  | - | - | - | - | - | - | - | - | - | - | - | - | - | #6 | *Africa 1* |
| GAB3-2007-GAL-DOM3 | Chicken | Libreville | [100-1000[ | 3/3 | 100 (3/3) | 7 |  | - | - | - | - | - | - | - | - | - | - | - | - | - | #6 | *Africa 1* |
| GAB3-2007-GAL-DOM4 | Chicken | Libreville | [100-1000[ | 3/3 | 100 (3/3) | 10-11 |  | - | - | - | - | - | - | - | - | - | - | - | - | - | #6 | *Africa 1* |
| GAB3-2007-GAL-DOM11 | Chicken | Libreville | ≥1000 | 3/3 | 100 (3/3) | 8 |  | - | - | - | - | - | - | 223 | 166 | 147 | 126 | 111 | 129 | 273 | #7 | *Africa 1* |
|  |  |  |  |  |  |  |  |  |  |  |  |  |  |  |  |  |  |  |  |  |  |  |
| GAB2-2007-GAL-DOM4 | Chicken | Makokou | [100-1000[ | 0/3 | 100 (3/3) | 13-19 |  | 291 (1) | 242 (3) | 207 (2) | 342 (1) | 160 (1 or 3) | 165(3) | 231 | 166 | 142 | 136 | 111 | 135 | 273 | #8 | *Africa 3* |
| GAB2-2007-GAL-DOM5 | Chicken | Makokou | ≥1000 | 0/3 | 100 (3/3) | 13-19 |  | - | - | - | - | - | - | - | - | - | - | - | - | - | #8 | *Africa 3* |
| GAB3-2007-GAL-DOM5 A | Chicken | Libreville | NA | 0/3 | NA | NA |  | - | - | - | - | - | - | 229 | 166 | 142 | 134 | 111 | 135 | 273 | #9 | *Africa 3* |
| GAB3-2007-GAL-DOM10 | Chicken | Libreville | ≥1000 | 3/3 | 100 (3/3) | 12-20 |  | - | - | - | - | - | - | - | - | - | - | - | - | - | #9 | *Africa 3* |
| GAB3-2007-GAL-DOM16 | Chicken | Libreville | ≥1000 | 3/3 | 100 (3/3) | 12-15 |  | - | - | - | - | - | - | - | - | - | - | - | - | - | #9 | *Africa 3* |
| GAB3-2007-GAL-DOM7 | Chicken | Libreville | [100-1000[ | 3/3 | 100 (3/3) | 13-17 |  | - | - | - | - | - | - | - | - | - | - | - | - | - | #9 | *Africa 3* |
| GAB3-2007-GAL-DOM9 | Chicken | Libreville | ≥1000 | 3/3 | 100 (3/3) | 9-16 |  | - | - | - | - | - | - | - | - | - | - | - | - | - | #9 | *Africa 3* |
| GAB3-2007-GAL-DOM6 | Chicken | Libreville | ≥1000 | 3/3 | 100 (3/3) | 14-17 |  | - | - | - | - | - | - | 227 | 166 | 142 | 136 | 111 | 135 | 275 | #10 | *Africa 3* |
| GAB3-2007-GAL-DOM8 | Chicken | Libreville | ≥1000 | 3/3 | 100 (3/3) | 13-20 |  | - | - | - | - | - | - |  |  |  |  |  |  |  | #10 | *Africa 3* |
| GAB5-2007-GAL-DOM4 | Chicken | Franceville | <100 | 3/3 | 100 (3/3) | 11-16 |  | - | - | - | - | - | - | 225 | 166 | 145 | 140 | 111 | 135 | 275 | #11 | *Africa 3* |
| GAB5-2007-GAL-DOM5 | Chicken | Franceville | [100-1000[ | 3/3 | 100 (3/3) | 9-12 |  | - | - | - | - | - | - | - | - | - | - | - | - | - | #11 | *Africa 3* |
| GAB5-2007-GAL-DOM6 | Chicken | Franceville | [100-1000[ | 3/3 | 100 (3/3) | 16-17 |  | - | - | - | - | - | - | - | - | - | - | - | - | - | #11 | *Africa 3* |
| GAB1-2007-GAL-DOM10 | Chicken | Dienga | ≥1000 | 0/3 | 100 (3/3) | 13-14 |  | - | - | - | - | - | - | 225 | 166 | 142 | 136 | 111 | 135 | 275 | #12 | *Africa 3* |
| GAB8-2007-GAL-DOM12 | Chicken | Mougoundou | ≥1000 | 2/3 | 100 (3/3) | 10-19 |  | - | - | - | - | - | - | - | - | - | - | - | - | - | #12 | *Africa 3* |
| GAB3-2007-GAL-DOM12 | Chicken | Libreville | ≥1000 | 1/3 | 100 (3/3) | 11-17 |  | - | - | - | - | - | - | 225 | 166 | 142 | 136 | 111 | 135 | 273 | #13 | *Africa 3* |
| GAB5-2007-GAL-DOM3 | Chicken | Franceville | <100 | 0/3 | 33 (1/3) | 12 |  | - | - | - | - | - | - | 225 | 166 | 142 | 132 | 111 | 135 | 271 | #14 | *Africa 3* |
| GAB2-2007-GAL-DOM1 | Chicken | Makokou | ≥1000 | 0/3 | 100 (3/3) | 16-22 |  | - | - | - | - | - | - | 223 | 166 | 142 | 136 | 111 | 135 | 279 | #15 | *Africa 3* |
| GAB2-2007-GAL-DOM3 | Chicken | Makokou | [100-1000[ | 0/3 | 100 (3/3) | 15-22 |  | - | - | - | - | - | - |  |  |  |  |  |  |  | #15 | *Africa 3* |
| GAB2-2007-GAL-DOM2 | Chicken | Makokou | ≥1000 | 0/3 | 100 (3/3) | 15-22 |  | - | - | - | - | - | - | 223 | 166 | 142 | 136 | 111 | 135 | 277 | #16 | *Africa 3* |
|  |  |  |  |  |  |  |  |  |  |  |  |  |  |  |  |  |  |  |  |  |  |  |
| GAB2-2007-GAL-DOM6 | Chicken | Makokou | ≥1000 | 0/3 | 0 (0/3) | Survived |  | 289 (2 or 3) | 242 (2) | 207(2) | 336 (2 or 3) | 160 (1 or 3) | 165(3) | 223 | 190 | 147 | 144 | 111 | 141 | 261 | #17 |  |
|  |  |  |  |  |  |  |  |  |  |  |  |  |  |  |  |  |  |  |  |  |  |  |
| GAB1-2007-CAP-AEG6 | Goat | Dienga | <100 | 0/3 | 0 (0/3) | Survived |  | 289 (2 or 3) | 242 (3) | 205 (3) | 336 (2 or 3) | 160 (1 or 3) | 165(3) | 213 | 190 | 149 | 128 | 111 | 137 | 267 | #18 | *Type III* |
| GAB1-2007-FEL-CAT1 | Cat | Dienga | [100-1000[ | 3^c^/6 | 50 (3/6) | 11-27 |  | - | - | - | - | - | - | - | - | - | - | - | - | - | #18 | *Type III* |
| GAB1-2007-GAL-DOM13 | Chicken | Dienga | <100 | 0/3 | 0 (0/3) | Survived |  | - | - | - | - | - | - | - | - | - | - | - | - | - | #18 | *Type III* |
| GAB1-2007-GAL-DOM16 | Chicken | Dienga | [100-1000[ | 0/3 | 0 (0/3) | Survived |  | - | - | - | - | - | - | - | - | - | - | - | - | - | #18 | *Type III* |
| GAB1-2007-GAL-DOM17 | Chicken | Dienga | ≥1000 | 0/3 | 0 (0/3) | Survived |  | - | - | - | - | - | - | - | - | - | - | - | - | - | #18 | *Type III* |
| GAB1-2007-GAL-DOM20 | Chicken | Dienga | ≥1000 | 3/3 | NA | NA |  | - | - | - | - | - | - | - | - | - | - | - | - | - | #18 | *Type III* |
| GAB1-2007-GAL-DOM9 | Chicken | Dienga | [100-1000[ | 0/1 | 0 (0/1) | Survived |  | - | - | - | - | - | - | - | - | - | - | - | - | - | #18 | *Type III* |
| GAB1-2007-OVI-ARI3 | Sheep | Dienga | <100 | 0/6 | 0 (0/6) | Survived |  | - | - | - | - | - | - | - | - | - | - | - | - | - | #18 | *Type III* |
| GAB1-2007-OVI-ARI6 | Sheep | Dienga | <100 | 0/5 | 0 (0/5) | Survived |  | - | - | - | - | - | - | - | - | - | - | - | - | - | #18 | *Type III* |
| GAB1-2007-OVI-ARI7 | Sheep | Dienga | <100 | 0/6 | 50 (3/6) | 17-23 |  | - | - | - | - | - | - | - | - | - | - | - | - | - | #18 | *Type III* |
| GAB7-2007-GAL-DOM7 | Chicken | Léconi | [100-1000[ | 3/3 | 100 (2/3) | 13-24 |  | - | - | - | - | - | - | 213 | 190 | 147 | 128 | 111 | 137 | 275 | #19 | *Type III* |
| GAB3-2007-GAL-DOM13 | Chicken | Libreville | <100 | 0/3 | 33 (1/3) | 18 |  | - | - | - | - | - | - | 213 | 190 | 147 | 126 | 111 | 139 | 265 | #20 | *Type III* |
| GAB1-2007-CAP-AEG1 | Goat | Dienga | <100 | 0/3 | 0 (0/3) | Survived |  | - | - | - | - | - | - | 213 | 190 | 147 | 126 | 111 | 137 | 273 | #21 | *Type III* |
| GAB1-2007-CAP-AEG3 | Goat | Dienga | <100 | 0/6 | 0 (0/6) | Survived |  | - | - | - | - | - | - | 213 | 190 | 145 | 128 | 111 | 137 | 273 | #22 | *Type III* |
| GAB1-2007-CAP-AEG5 | Goat | Dienga | <100 | 1/3 | 33 (1/3) | 12 |  | - | - | - | - | - | - | - | - | - | - | - | - | - | #22 | *Type III* |
| GAB1-2007-CAP-AEG9 | Goat | Dienga | <100 | 0/4 | 0 (0/4) | Survived |  | - | - | - | - | - | - | - | - | - | - | - | - | - | #22 | *Type III* |
| GAB1-2007-GAL-DOM11 | Chicken | Dienga | [100-1000[ | 0/2 | 0 (0/2) | Survived |  | - | - | - | - | - | - | - | - | - | - | - | - | - | #22 | *Type III* |
| GAB1-2007-OVI-ARI4 | Sheep | Dienga | <100 | 0/1 | 0 (0/1) | Survived |  | - | - | - | - | - | - | - | - | - | - | - | - | - | #22 | *Type III* |
| GAB1-2007-GAL-DOM5 | Chicken | Dienga | <100 | 0/2 | 0 (0/2) | Survived |  | - | - | - | - | - | - | 213 | 190 | 145 | 128 | 111 | 137 | 271 | #23 | *Type III* |
| GAB1-2007-CAP-AEG2 | Goat | Dienga | <100 | 0/5 | 0 (0/5) | Survived |  | - | - | - | - | - | - | 211 | 190 | 147 | 124 | 111 | 137 | 267 | #24 | *Type III* |
| GAB1-2007-CAP-AEG7 | Goat | Dienga | <100 | 0/3 | 0 (0/3) | Survived |  | - | - | - | - | - | - | - | - | - | - | - | - | - | #24 | *Type III* |
| GAB1-2007-CAP-AEG8 | Goat | Dienga | <100 | 0/6 | 0 (0/6) | Survived |  | - | - | - | - | - | - | - | - | - | - | - | - | - | #24 | *Type III* |
| GAB1-2007-GAL-DOM1 | Chicken | Dienga | ]100-1000] | 0/3 | 0 (0/3) | Survived |  | - | - | - | - | - | - | - | - | - | - | - | - | - | #24 | *Type III* |
| GAB1-2007-GAL-DOM14 | Chicken | Dienga | ]100-1000] | 0/3 | 0 (0/3) | Survived |  | - | - | - | - | - | - | - | - | - | - | - | - | - | #24 | *Type III* |
| GAB1-2007-GAL-DOM15 | Chicken | Dienga | ]100-1000] | 0/3 | 0 (0/3) | Survived |  | - | - | - | - | - | - | - | - | - | - | - | - | - | #24 | *Type III* |
| GAB1-2007-GAL-DOM18 | Chicken | Dienga | ≥1000 | 0/2 | 0 (0/2) | Survived |  | - | - | - | - | - | - | - | - | - | - | - | - | - | #24 | *Type III* |
| GAB1-2007-GAL-DOM2 | Chicken | Dienga | ≥1000 | 0/3 | 0 (0/3) | Survived |  | - | - | - | - | - | - | - | - | - | - | - | - | - | #24 | *Type III* |
| GAB1-2007-GAL-DOM6 | Chicken | Dienga | <100 | 0/2 | 0 (0/2) | Survived |  | - | - | - | - | - | - | - | - | - | - | - | - | - | #24 | *Type III* |
| GAB1-2007-GAL-DOM7 | Chicken | Dienga | ]100-1000] | 0/3 | 0 (0/3) | Survived |  | - | - | - | - | - | - | - | - | - | - | - | - | - | #24 | *Type III* |
| GAB1-2007-GAL-DOM8 | Chicken | Dienga | <100 | 0/3 | 0 (0/3) | Survived |  | - | - | - | - | - | - | - | - | - | - | - | - | - | #24 | *Type III* |
| GAB1-2007-OVI-ARI1 | Sheep | Dienga | <100 | 0/5 | 0 (0/5) | Survived |  | - | - | - | - | - | - | - | - | - | - | - | - | - | #24 | *Type III* |
| GAB1-2007-OVI-ARI2 | Sheep | Dienga | <100 | 0/5 | 0 (0/5) | Survived |  | - | - | - | - | - | - | - | - | - | - | - | - | - | #24 | *Type III* |
| GAB1-2007-OVI-ARI5 | Sheep | Dienga | ]100-1000] | 0/6 | 0 (0/6) | Survived |  | - | - | - | - | - | - | - | - | - | - | - | - | - | #24 | *Type III* |
| GAB1-2007-GAL-DOM4 | Chicken | Dienga | ]100-1000] | 0/3 | 0 (0/3) | Survived |  | - | - | - | - | - | - | - | - | - | - | - | - | - | #24 | *Type III* |
| GAB1-2007-GAL-DOM3 | Chicken | Dienga | ]100-1000] | 0/3 | 0 (0/3) | Survived |  | - | - | - | - | - | - | 211 | 190 | 147 | 124 | 111 | 137 | 265 | #25 | *Type III* |
|  |  |  |  |  |  |  |  |  |  |  |  |  |  |  |  |  |  |  |  |  |  |  |
| GAB4-2007-GAL-DOM1 | Chicken | La Lopé | [100-1000[ | 0/2 | 0 (0/2) | Survived |  | 291 (1) | 242 (3) | 205 (3) | 336 (2 or 3) | 160 (1 or 3) | 165(3) | 213 | 190 | 145 | 128 | 111 | 131 | 269 | #26 | *Type III like* |
| GAB1-2007-CAP-AEG10 | Goat | Dienga | <100 | 0/2 | 0 (0/2) | Survived |  | - | - | - | - | - | - | 211 | 190 | 147 | 124 | 111 | 137 | 267 | #27 | *Type III like* |
| GAB1-2007-CAP-AEG4 | Goat | Dienga | <100 | 0/2 | 50 (1/2) | 22 |  | - | - | - | - | - | - | - | - | - | - | - | - | - | #27 | *Type III like* |
|  |  |  |  |  |  |  |  |  |  |  |  |  |  |  |  |  |  |  |  |  |  |  |

Note: - : Identical to the previous value (located above).

^a^: GAB (Gabon), 1(Dienga), 2 (Makokou), 3 (Libreville), 4 (La Lopé), 5 (Franceville) – year of isolation – abbreviation of Latin name of the species (*Gallus domesticus, Ovis aries, Capra aegagrus hircus, Felis catus*).

^b^ Number of parasites estimated from semi-quantitative Real-time PCR assays on 200 µl of animal tissue preparation.

^c^ Only one out of the three mice with ascitic fluid survived.

^d^ Allelic polymorphism of MS markers are expressed as sizes of PCR products (bp). ( ) Allelic polymorphism of markers *TUB2, W35, TgM-A, B18, B17* and *M33* are expressed as numbers relative to classic typing of *T. gondii*: alleles 1, 2, 3 are reserved for clonal lineages I, II and III; allele 1 or 3 means that Types I and III share the allele; allele 2 or 3 means that Types II and III share the allele.

^e^ Numbers relative to classic typing were adjusted after sequencing of W35 marker.
